# Socioeconomic inequalities in the continuum of care across women’s reproductive life cycle in Bangladesh

**DOI:** 10.1038/s41598-022-19888-w

**Published:** 2022-09-16

**Authors:** Nadira Parvin, Mosiur Rahman, Md. Jahirul Islam, Syed Emdadul Haque, Prosannajid Sarkar, Md. Nazrul Islam Mondal

**Affiliations:** 1 Department of Population Science and Human Resource Development, University of Rajshahi, Rajshahi, 6205 Bangladesh; 2 Griffith Criminology Institute, Griffith University, Brisbane, QLD 4122 Australia; 3 Country Representative, University of Chicago Research, Dhaka, Bangladesh; 4 Dr. Wazed Research and Training Institute, Begum Rokeya University, Rangpur, 5404 Bangladesh

**Keywords:** Health care, Public health, Epidemiology

## Abstract

We examined the association between socioeconomic status (SES) and continuum of care (CoC) completion rate in maternal, neonatal, and child health among mothers in Bangladesh. We used data from the Bangladesh Demographic Health Survey (BDHS) from 2017 to 2018. Our findings were based on the responses of 1527 married women who had at least one child aged 12 to 23 months at the time of the survey. As a measure of SES, we focused on the standard of living (hereinafter referred to as wealth). The CoC for maternal and child health (MNCH) services is the study's outcome variable. The CoC was calculated using seven MNCH interventions: four or more antenatal care (ANC) visits with a skilled practitioner, delivery by a skilled birth attendant, post-natal care for mothers (PNCM) within two days of giving birth, post-natal care for newborns (PNCM) within two days of birth, immunization, age-appropriate breastfeeding, and maternal current use of modern family planning (FP) methods. Only 18.1% of Bangladeshi women completed all seven MNCH care interventions during the reproductive life span. Participants in the high SES group were 2.30 times (95% confidence interval [CI] 1.61–3.28) more likely than those in the low SES group to have higher composite care index (CCI) scores. Women with secondary or higher secondary education, as well as women who were exposed to mass media at least once a week, women who lived in an urban setting, women who had an intended pregnancy, and women with one parity, are associated with high CCI scores when other sociodemographic variables are considered. The complete CoC for MNCH reveals an extremely low completion rate, which may suggest that Bangladeshi mothers, newborns, and children are not receiving the most out of their present health care. Participants in the high SES group displayed higher CCI values than those in the low SES group, indicating that SES is one of the primary drivers of completion of CoC for MNCH services.

## Introduction

Despite substantial progress in reducing the maternal mortality ratio (MMR) from 550 per 100,000 live births in 1990 to 176 per 100,000 live births in 2015, Bangladesh's MMR remains notably high compared to other developing countries^1^. To reach the Sustainable Development Goals (SDGs) 3.1, the country must reduce MMR to fewer than 70 per 100,000 live births by 2030^2^. Bangladesh's average annual rate of MMR reduction was only 5% from 1990 to 2015^3^. If Bangladesh aims to meet the MMR target of the SDGs, a reduction in maternal fatalities is critical for progress.

While Bangladesh accomplished the Millennium Development Goal-4 (MDG-4 goal) of lowering the under-five mortality rate to 46 per 1000 live births, progress in lowering the neonatal mortality rate (NMR) has been slower. For example, between 1990 and 2015, the post-neonatal under-five mortality rate fell by 71%, whereas the NMR fell by only 46%^4^. In 2014, the neonatal mortality in Bangladesh accounted for more than 60% of all deaths among children under the age of five^4^ Accelerating the rate of progress toward reducing neonatal mortalities is therefore important in order to accomplish the NMR goal of the SDGs in this country.

At least four visits of antenatal care (ANC), delivery by a skilled birth attendant (SBA), and postnatal care (PNC) for mothers have all been demonstrated to help reduce the MMR^5,6^. In Bangladesh, however, only around 47% of pregnant women received four or more ANC visits (ANC 4+), only 53% of births were supported by SBA, and only 52% of women received PNC within two days after delivery for their most recent birth^7^. Furthermore, PNC for newborns, immunization coverage, and age-related breastfeeding practice^8,9^ have all been demonstrated to be effective strategies for lowering neonatal and child mortality. According to 2017–18 Bangladesh Demography and Health Survey (BDHS) survey, 46% of neonates were not given any PNC^7^.

Given the significance of these indicators, they are currently being evaluated for consistency to ensure the mother's and newborn's survival and well-being. The comprehensive continuum-of-care (CoC) approach, which defines access to comprehensive, integrated, and continuous interventions throughout the life cycle (pregnancy, delivery, postnatal, infancy, childhood, and reproductive health period), may be a crucial strategy for decreasing the maternal and newborn mortality rate and meeting the SDGs’ target of halving maternal and newborn deaths^10^.

While data on the proportion of women and children in Bangladesh who receive individual maternal and child health (MNCH) care services such as ANC visits, SBA deliveries, PNC for mothers and newborns, childhood immunization, and modern FP methods is available, there is no clear data on the proportion of women who drop out or do not complete the CoC throughout their reproductive life cycle. Completion of a singular MNCH service without the completion or follow-through of other set of interventions will not guarantee that the women receive a comprehensive set of beneficial outcomes throughout the life cycle. As a result, a better understanding of where the gaps lie in access to treatment and the variables that mediate this is necessary for a successful implementation of improved CoC for MNCH services.

Despite the fact that mothers, newborns, and children are intimately linked in life and healthcare requirements^11^, women and children have historically been treated separately in MNCH policies and programs^11^, resulting in healthcare gaps. The majority of existing research in Bangladesh as well as other low-resource settings focused on the continuity of health care services provided to women during pregnancy, delivery, and the postnatal period^12–16^. However, literature that concerns the CoC for women from pregnancy to childhood is scarce. Two research studies on CoC care for women from pregnancy to childhood have been found^17,18^. A study conducted in the Gambia, for example, used Demographic and Health Survey data to analyze the factors associated with the continuum of maternal, newborn, and child health care^17^. Another cross-sectional study in Ghana looked at individual and community-level characteristics linked to CoC attainment from pregnancy to childhood^18^.

Lack of education^13,14^, low service accessibility^16^, high birth order^12,13,15^, unemployment^13,16^, unintended pregnancies^14^, rural residence^13,15^, access to media^15^ and women empowerment^13^ are thought to be major determinants of low rates of completion of the CoC among during pregnancy, delivery, and the postnatal period in Bangladesh and other low resource settings. Furthermore, prior studies from Gambia^17^ and Ghana^18^ found that women's autonomy, respondents' and husbands' education, media exposure, high birth order, traditional religious practices, and area of residence were all linked to the continuum of maternity, newborn, and child health care. However, in the completion of the CoC for maternal health or the comprehensive CoC from pregnancy to childhood, factors such as socioeconomic status (SES) has received little consideration in literature.

Bangladesh is undergoing a rapid economic and epidemiologic shift, with increased SES inequality as a result of industrialization and urbanization^19^. As a result, a focus on the relationship between SES inequality and the continuum of maternity, newborn, and child health care is required to reach progress toward the MMR and NMR goals of the SDGs in Bangladesh. Based on these concerns, the goals of this study were to: i) examine the level of CoC from pregnancy through childhood, as well as the reproductive healthcare period; and ii) determine the relationship between SES and maternal and child health (MNCH) care continuity.

## Methods

### Data source and sample

The Bangladesh Demographic and Health Survey (BDHS), a nationally representative household-based survey conducted in 2017–2018^7^, provided the data for this study. In the 2017–2018 BDHS, a two-stage sampling design was adopted. In the first stage, 675 primary sampling units (PSU) were established (urban areas: 250; rural areas: 425). The PSU was created using data from the 2011 Bangladeshi census. Each PSU was assigned a systematic sample of 30 households on average in the second stage. The survey was completed in 672 clusters after three clusters (one urban and two rural) were completely degraded by floodwater. A total of 20,160 households were surveyed. To eliminate bias, no substitutions or changes to the pre-selected households were allowed. The BDHS gathered information from ever-married women of reproductive age (15–49 years) who stayed in the selected houses the night before the survey^7^.

Six questionnaires were employed in the study: one for the household, another for women, a third for biomarkers, a fourth for two verbal autopsies, a fifth for the community, and a sixth for the fieldworker. The women's questionnaire provided the data for the selected MNCH indicators. The questionnaires were first developed in English and then translated into Bangla, Bangladesh's official language. The questionnaire's reliability was determined through a pilot study^7^. To capture demographic information about the household and its members, skilled data collectors performed face-to-face interviews with an adult member of each of the 20,160 randomly selected households, resulting in a household response rate of 99%. Ninety nine percent of the 20,376 women in those households who were eligible completed a survey on MNCH behaviors and outcomes.

**Figure 1:**
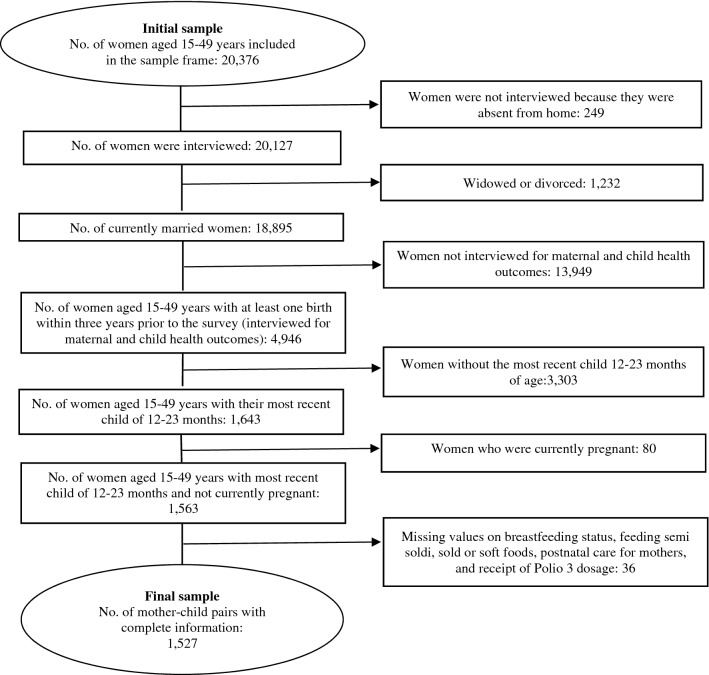
Selection of the sample: Bangladesh Demographic and Health Survey 2017–2018.

We selected women between the ages of 15 and 49 who had given birth in the three years prior to the survey in our sample since data on ANC, delivery assistance, and PNC were only collected for the most recent live birth in the three years before to the survey in the 2017–2018 BDHS. To determine the rate of modern contraceptive use, one of the parts of the continuity of care, we chose non-pregnant women. Because all four vaccinations are recommended for children under the age of 12 months in Bangladesh, women with children under the age of 12 months or over the age of 23 months were excluded for calculating the child's vaccination coverage. The study's final analytical sample consisted of 1527 married women aged 15–49 years old, with their most recent children ranging in age from 12 to 23 months at the time of the survey (Fig. 1).

### Outcome measures

The CoC for MNCH services was the study's outcome variable. Instead of focusing on the usage of each MNCH care, the composite coverage index (CCI) was employed to assess CoC. The indicators used to measure CCI were chosen based on the following criteria: (1) they were recommended by international guidelines^6,10,17^; (2) they were linked to significant health outcomes^5,6,8,9^; and (3) they were included in the 2017–2018 BDHS survey^7^.

Based on Kerber et al.^10^ descriptions of the CoC and World Health Organization’s (WHO)^20^ recommended MNCH care, we implemented the notion of CCI with certain modifications. Eight interventions are included in the original CCI^20^. The interventions are as follows: (i) FP coverage; (ii) SBA; (iii) at least one ANC visit by a skilled provider; (iv) bacille Calmette–Guérin (BCG) vaccination; (v) three diphtheria–tetanus–pertussis (DTP3) vaccinations; (vi) measles (MSL) vaccination; (vii) oral rehydration therapy for infant diarrhea; and (viii) care-seeking for childhood pneumonia. In this study, modified CCI entails following the stages of the CoC: pregnancy, delivery, PNC, newborn care, childhood, and reproductive health.

CCI was calculated using seven essential MNCH interventions: (1) maternal health care (ANC ≥ 4), SBA, and PNC for mothers within two days after birth), (2) newborn health care (PNC within two days after birth), (3) child health care (immunization and AABF), and (4) reproductive health care (FP).

Four or more visits to ANC with a skilled practitioner (doctor, nurse, midwife, or auxiliary nurse) are referred to as ANC ≥ 4 visits; ANC ≥ 4 visits were defined as pregnancy-level CoC. The acronym SBA stands for skilled birth attendant. Acquiring SBA such as qualified doctors, nurses, midwives, or paramedics, family welfare visitors (FWVs), community skilled birth attendants (CSBAs), and sub-assistant community medical officers (SACMOs) at delivery were considered to be CoC at the stage of delivery. PNCM denotes mothers who had their first health check within two days of giving birth. Newborns who had their initial health check within two days of birth are referred to as PNCN.

The acronym AABF stands for age-appropriate breastfeeding. Breastfeeding should be continued with appropriate complementary and solid, semisolid, or soft foods for children aged 12–23 months^21^. AABF was completed by women who continued to breastfeed their children at the time of the survey and were given solid, semi-solid, or soft foods 24 h prior to the study. FP is for family planning, and it refers to women who are currently utilizing (or whose partners are using) modern family planning methods at the time of the survey. One dose of BCG, three doses of DPT3, three doses of poliomyelitis vaccine (PL), and one dose of MSL were used to assess childhood immunization.

The level of CoC is calculated with the following formula:

$${\mathrm{CCI}}= \frac{1}{7}({\mathrm{ANC}}\ge 4+{\mathrm{SBA}}+{\mathrm{PNCM}}+{\mathrm{AABF}}+{\mathrm{PNCN}}+\frac{{\mathrm{BCG}}+2{\mathrm{DPT}}3+2{\mathrm{PL}}+{\mathrm{MSL}}}{6}+{\mathrm{FP}})$$

Each stage is given the same weight, and the indicators within each stage are given equal weights, with the exception of DTP3 and PL, which are given two weights because they require more than one dose. The scores obtained were then recoded as terciles with the categories labeled as low, middle, and high CCI.

### SES

As a measure of SES, the wealth index was used in this study. This survey's wealth index was produced using data on household assets, such as durable goods ownership, home characteristics, and building materials^7^. Each asset was given a weight (factor score) using principal component analysis. Each asset was then assigned a score to each household. After that, the sample was divided into population terciles, with each household allocated to the low, medium, or high tercile, and women were ranked based on the total score of the household in which they lived.

### Covariates

The variables for this research were chosen based on their theoretical and empirical significance in various types of literature^10–18^. The ages of the mothers were separated into three categories: 15–24 years (youngest), 25–34 years (middle), and 35–49 years (oldest). The tercile was used to determine parity. A dichotomous variable was created to quantify pregnancy intentions (intended: live birth wanted at the time of conception or unintended: live birth wanted after conception or not wanted at all). The total number of household members was categorized using tercile. The location of residence was classified as either rural or urban. Exposure to the mass media was determined by watching television, listening to the radio, or reading the newspaper at least once a week.

The educational level of women and husbands was determined using Bangladesh's formal education system: no education (0 years), primary (1–5 years), secondary (6–10 years), or higher (11 years or more). We examined whether a woman participated in obtaining health care for herself alone or jointly to assess women's decision-making autonomy in their own health care. The mother's occupation was categorized based on whether she was unemployed or employed in a manual, nonmanual, or agricultural occupation. To distinguish between male and female offspring, a binary variable was constructed.

### Statistical analyses

The data was extracted, cleaned, recoded, and analyzed using the STATA version 14.1 software. The analysis used weighting to adjust for differential selection probabilities due to the BDHS data sampling approach. This research looked at descriptive statistics for the sample's background characteristics. Descriptive data on the rate of utilization of important MNCH services during the reproductive life cycle of women were also supplied along the continuum. To quantify distinct dimensions of SES inequalities in the CCI scores, we employed three regression-based methods: (1) relative index of inequality (RII), (2) slope index of inequality (SII), and (3) adjusted odds ratio (AOR). The RII and SII are regression-based inequality measures that consider CCI scores throughout the whole socioeconomic distribution in the study population, whereas the AOR simply examines relative disparities in CCI scores between the wealthiest and most disadvantaged groups^22,23^.

The SII was calculated using linear regression, with the relative rank of the socioeconomic measurement factor serving as a predictor variable and the CCI scores serving as an outcome variable. A negative SII suggests that the unfavorable health indicator drops as SES rises, whereas a positive SII shows that the health indicator rises as SES grows; a value of 0 indicates that there is no relationship between adverse health and SES^24^. The cumulative proportion of the sample in each social class group was computed for the relative rank of social class. The coding for the various socioeconomic classes was based on the midpoint. For example, the low social class category in our sample data included 32.8% of the individuals and was assigned the value of 0.164 (32.8/2), the middle social class category included 34.4% of the individuals and was assigned the value of 0.50 (0.328 + [0.344/2]), and the high social class category included 32.8% of the individuals and was assigned the value of 0.84 (1−[0.328/2])^24^. We used regression diagnostics to see if our data satisfied the regression analysis assumption before computing the linear regression. We tested the following assumptions in particular: normality and collinearity. Kernel density plots and histograms were used to assess for normality in the residuals. We determined that residuals were normally distributed based on the results of those experiments. The variance inflation factors were used to test multicollinearity (VIF). No variable had a VIF of more than ten or a 1/VIF of less than 0.1.

We used a modified robust Poisson technique based on semiparametric theory to compute RII because our data did not meet the well-known property of the Poisson distribution, which is that it compels the dispersion to be equal to the mean, as advised by Zou^25^. This viewpoint emphasizes that the robust Poisson technique does not require the outcome to have a Poisson distribution. When the RII is less than 1, it means that the low SES are more likely than the high SES to have low CCI scores^26^.

The association between the SES and the CCI scores, namely low, medium, and high CCI scores, were investigated using adjusted ordinal logistic analysis using low CCI scores as the reference level. Brant's test^27^ revealed that our ordinal regression model's proportionate odds assumption was not broken. In the multiple regression models, independent variables were included at the same time. Point bi-serial correlation coefficients were employed to assess the possibility of collinearity among the independent variables. Due to substantial correlations with maternal education level (r = 0.59), the husband's education was not included in any adjusted analysis. To analyze the strength of the associations, we calculated the odds ratios and used the 95% confidence intervals (CIs) for significance testing. For all analyses, the significance level was fixed at *P* < 0.05.

### Ethical considerations

The BDHS data collection techniques were authorized by both the ICF's Independent Review Board (IRB) and Bangladesh's National Ethics Committee. After hearing about the survey's objectives, each respondent gave their informed consent. The permission form stated the study's goal as well as the confidentiality of the interviews, and the respondents' rights to freely engage in the study and to withdraw at any time without consequence. Because it was based on public use of a secondary data collection that was anonymous with no identifying information on the survey participants, this study was exempted from full review. All study procedures were carried out in conformity with the principles of the 2013 revision of the Declaration of Helsinki.

## Results

### Background characteristics

Table 1 summarizes the background characteristics of the participants in this investigation. The study looked at 1527 women who had at least one child between the ages of 12 and 23 months. The majority of the participants were between the ages of 20 and 34. The majority of the women lived in rural areas, 22.6% of the births were unplanned, and 30.4% of the sample were ≥ 3 parity women.

**Table 1:** Characteristics of the study population: Bangladesh Demographic and Health Survey 2017–18 (n = 1527).

Characteristics	Number^a^	Percentage^b^ (95% CI)
**Age, y**		
15–24	783	52.4 (49.7–55.2)
25–34	632	40.4 (37.8–43.0)
35–49	112	7.2 (5.9–8.7)
**Education**		
No education	89	5.8 (4.6–7.4)
Primary	425	28.2 (25.6–31.0)
Secondary	721	47.5 (44.7–50.2)
Higher	292	18.5 (16.3–20.9)
**Husband’s education**		
No education	202	13.1 (11.0–15.5)
Primary	543	35.7 (32.8–38.6)
Secondary	486	32.6 (29.7–35.5)
Higher	296	18.7 (16.4–21.2)
**Decision-making autonomy, own health care**^**c**^		
No	416	28.1 (25.5–30.9)
Yes	1111	71.9 (69.1–74.6)
**Respondent employed**		
No	949	62.0 (58.9–65.0)
Yes	578	38.0 (35.0–41.2)
**Mass media exposure**^**d**^		
No	509	31.9 (28.9–35.2)
Yes	1018	68.1 (64.8–71.2)
**Parity (tercile)**		
1	557	36.3 (33.7–38.9)
2	503	33.4 (30.8–36.1)
≥ 3	467	30.4 (27.8–33.1)
**Pregnancy intended**^**e**^		
No	348	22.6 (20.3–25.0)
Yes	1179	77.4 (75.0–79.7)
**No. household member (tercile)**		
2–4	477	31.8 (29.2–34.7)
5–6	563	36.5 (33.7–39.3)
≥ 7	487	31.7 (28.8–34.7)
**Place of residence**		
Rural	1005	74.0 (71.0–76.0)
Urban	522	26.0 (24.0–29.0)
**Offspring sex**		
Female	747	49.6 (46.9–52.3)
Male	780	50.4 (47.7–53.2)
**SES**		
Low	509	32.8 (29.7–36.1)
Middle	509	34.4 (31.5–37.6)
High	509	32.8 (29.6–36.1)

CI = confidence interval. ^a^Numbers are unweighted; ^b^Percentages are weighted; ^c^women participated alone or jointly in the decision making on own health care; ^d^watching television, listening to the radio, or reading the newspaper at least once a week; ^e^Intended: live birth wanted at time of conception or unintended: live birth wanted after conception or not wanted at all.

Around 72% of women decided on their own healthcare, 5.8% of women had no education, and 13.1% of their husbands or partners had no education. In our sample data, 68.1% of the respondents were exposed to the media at least once a week, and nearly half of the women's last child (50.4%) was a male child. Regarding participants’ SES 67.2% of the women were in the middle and high SES groups.

### MNCH care coverage and continuum-of-care achievement

The MNCH care coverage along the CoC is shown in Table 2. About 309 (18.1%) of 1527 women completed all phases of CoC during their reproductive lives. Approximately 47% of women had four or more ANC visits, as indicated. A total of 53.3% of all deliveries were aided by the SBA. Almost half of all women and their newborns visited a PNC within the first 48 h after giving birth.

**Table 2:** Percent distribution of MNCH care coverage and CoC achievement: Bangladesh Demography and Health Survey 2017–2018 (n = 1527).

Life cycle	Care components	Number^a^	Percentage (95% CI)^b^
**Maternal health**			
Pregnancy	ANC ≥ 4	744	46.9 (43.7–50.2)
Delivery	SBA	824	53.3 (50.0–56.6)
Postnatal care	PNCM within two days after birth	764	49.4 (46.2–52.6)
**Newborn health**			
Postnatal care	PNCN within two days after birth	762	49.3 (46.1–52.5)
**Child health**			
Immunization	One dosage of BCG	1505	98.6 (97.5–99.2)
	Three dosages of DPT	1469	96.2 (94.8–97.2)
	Three dosages of Polio	1503	98.6 (97.5–99.2)
	One dosage of Measles	1379	90.5 (88.4–92.3)
Breast feeding	AABF	1410	91.4 (89.6–93.0)
**Reproductive health**			
Family planning	Current use of modern contraceptive method	1124	72.3 (69.4–75.1)
Complete CoC along the life cycle		309	18.1 (16.0–20.5)

CI = confidence interval. ^a^Numbers are unweighted; ^b^Percentages are weighted.

The majority of the mothers stated that their child, aged 12–23 months, had completed the prescribed BCG, DPT, Polio, and measles doses. BCG and three dosages of Polio immunization coverage were nearly 100% among the four vaccines. The AABF percentage was discovered to be 91.4%. A modern method of FP was utilized by 72.3% of women or their husbands.

**Figure 2:**
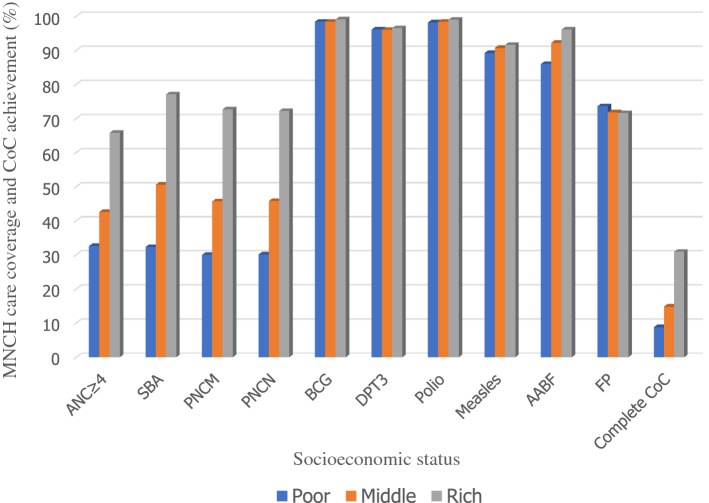
Percentage distribution of MNCH care coverage and CoC achievement by SES.

Figure 2 depicts the MNCH coverage and CoC achievement according to the SES. In comparison to women in the middle or high SES groups, women form low SES were less likely to receive all MNCH interventions as well as comprehensive CoC throughout their lives, except for the use of modern FP methods.

### Summary measures of SES inequality

Inequality measures are summarized in Table 3. An increase from the low to the high in SES distribution is associated with a 20% rise in high CCI scores, according to RII 1.20. SII indicates that the one-unit change from the low to the high of the SES group is associated with a 0.52-unit increase in the high CCI scores.

**Table 3:** Summary measures of SES and the CoC across the women’s reproductive life cycle: Bangladesh Demographic and Health Survey 2017–18 (n = 1527).

Inequality measure	Relative index of inequality (RII)		Slope index of inequality (SII)	
	RR (95% CI)	*P*-value	*ß* coefficient (95% CI)	*P*-value
SES	1.20 (1.11–1.29)	< 0.001	0.52 (0.31–0.74)	< 0.001

Note: CI = confidence interval; RR = risk ratio.

### Association between CCI scores and SES and other covariates

The findings of the ordinal logistic regression model evaluating the relationship between CCI scores, and SES and other factors are shown in Table 4. When compared to the low SES group, participants in the high SES group were 2.30 times (95% confidence interval [CI] 1.61–3.28) more likely to have higher CCI scores. Because the proportional odds assumption was upheld, the results produced by modeling medium CCI scores against low CCI values were interpreted in the same way.

**Table 4:** Ordinal logistic regression models for the association between SES and CoC across the women’s reproductive life cycle and other covariates: Bangladesh Demographic and Health Survey 2017–18 (n = 1527).

Characteristics	CCI scores	
	AOR (95% CI)	*P*-value
**Age, y (ref = 15–24)**		
25–34	1.20 (0.87–1.64)	0.270
35–49	1.39 (0.81–2.37)	0.232
**Education (ref = no education)**		
Primary	1.68 (0.97–2.88)	0.062
Secondary	2.81 (1.66–4.74)	< 0.001
Higher	6.50 (3.49–11.10)	< 0.001
**Decision-making autonomy, own health care (ref = no)**		
Yes	1.24 (0.97–1.57)	0.082
**Respondent employed (ref = no)**		
Yes	0.90 (0.71–1.15)	0.417
**Mass media exposure (ref = no)**		
Yes	1.73 (1.32–2.28)	< 0.001
**Parity (ref = 1)**		
2	0.65 (0.46–0.90)	0.009
≥ 3	0.50 (0.33–0.75)	0.001
**Pregnancy intended (ref = no)**		
Yes	1.35 (1.03–1.76)	0.029
**No. household member (ref = 2–4)**		
5–6	1.24 (0.93–1.66)	0.146
≥ 7	1.03 (0.76–1.39)	0.859
**Place of residence (ref = rural)**		
Urban	1.34 (1.02–1.74)	0.033
**Offspring sex (ref = female)**		
Male	1.06 (0.84–1.33)	0.640
**SES (ref = Low)**		
Middle	1.27 (0.94–1.71)	0.121
High	2.30 (1.61–3.28)	< 0.001

CI = confidence interval; AOR = adjusted odds ratio.

In terms of other factors, women with a secondary or higher secondary education, as well as women who were exposed to mass media at least once a week, were more likely than their peers to have high CCI scores. When compared to their counterparts, participants who lived in an urban location and had an intended pregnancy were 1.34 (95% CI 1.02–1.74) and 1.35 (95% CI 1.03–1.76) times more likely to have high CCI scores. In comparison to one parity women, 2 or ≥ 3 party women were associated with a decreased odd of higher CCI scores.

## Discussion

This is the first study to look at data from a national survey in Bangladesh to see how the CoC for maternal, neonatal, and child health relates to SES. Three major conclusions can be drawn: First, our research found very low level of CoC from pregnancy to childhood, with just 18.1% of Bangladeshi women completing seven MNCH care interventions along the continuum. Second, in comparison to the low SES group, participants in the high SES group had higher CCI scores. Finally, when other sociodemographic variables were considered, women with a secondary or higher secondary education, as well as women who were exposed to mass media at least once a week, women who lived in an urban setting, women who had an intended pregnancy, and women with one parity, had an increased likelihood of having higher CCI scores.

Despite Bangladesh's international praise for increasing MNCH services in line with the MDGs^14^, we found low completion rates for the comprehensive CoC for MNCH services. Furthermore, existing MNCH service use only contributes minimally to the SDG targets of universal healthcare coverage for improving unfavorable MNCH outcomes in Bangladesh. The low CoC rate for MNCH found in this investigation was comparable with what was observed in Gambia^17^ and Ghana^18^. Our findings are also consistent with earlier studies in Bangladesh^13,14^ that reported a poor completion rate for the entire CoC for maternal health.

Social disparities in health are well-known and diminishing social position has been linked to a stepwise or linear decline in health^28^. According to earlier research conducted in various low and middle-income countries (LMIC)^15,29,30^, which includes Bangladesh^13,14^, socioeconomic inequalities exist for the continuity of health care services offered to women during pregnancy, delivery, and the postnatal period. In keeping with these findings, our research found that participants from low SES had a higher likelihood of having lower CCI scores. Bangladesh allocates only 2.9% of its gross domestic product (GDP) to health spending, resulting in a high out-of-pocket health cost of 67%, one of the highest in the world^31^. Low SES groups are not able to afford the additional cost of health care, which may be one of the key reasons for such disparities.

Low SES mother–child pairings have a lower prevalence of CCI scores, which could be attributed to a lack of awareness and comprehension of the benefits of using comprehensive health care services during pregnancy, delivery, postnatal care, newborn care, childhood, and reproductive health^13^. It is also likely that women with a high socioeconomic status have more resources (such as money, automobiles, or motorcycles)^32^ and are more exposed to relevant MNCH information that can help them seek necessary MNCH interventions. Furthermore, some previous studies conducted in Bangladesh^33^ and in other neighboring countries such as India^34^ and Nepal^30^ stated that women from high socioeconomic groups have greater access to conventional health care facilities (such as hospitals or clinics), which could explain Bangladesh's higher MNCH CoC rate among those group.

Higher CCI scores of MNCH care were linked to women's secondary or higher educational levels, according to this study. Our findings are in line with earlier studies undertaken in a variety of developing countries^12,15,16,29,30,35^, including Bangladesh^13,14^, which found that women with higher education have better access to health care facilities during pregnancy, delivery, and the postpartum period. Our findings are also consistent with previous research in the Gambia^17^, which found that a woman's educational level is a significant determinant in completing CoC for MNCH care. One possible explanation is that educated women are more aware of the health benefits of accessing maternity care during pregnancy, childbirth, and postpartum, as well as newborn and child health care^13,14^. Other possible factors include the fact that education increases female autonomy and empowers women to make decisions about their own and their children's health^13^, the distribution of household resources, and the likelihood of getting written information about MNCH services.

Various studies have shown that media exposure influences CoC for maternal health service consumption^13,15^. In line with this previous research, the outcomes of the study imply that exposure to the media has a positive impact on the completion of CoC for MNCH care. More than 62% of Bangladeshis reside in rural areas, and nearly two-thirds of births take place there^36^. This study demonstrated that women residing in rural areas exhibited a negative connection with CoC completion for MNCH care, which was consistent with an earlier study in the Gambia^17^. This is likely due to a lack of travel time, poor transportation, and associated costs, which have been identified as key determinants of MNCH care utilization in Bangladesh^32,37,38^ and other low-resource contexts^30,39^.

Previous literautre^32,39,40^ has shown that women with more children receive fewer MNCH services and care. This could explain why some women believe that because they have had children before, they do not require healthcare services for higher-order births. In keeping with prior research, this study found that women with more children had a lesser chance of having high CCI scores. The findings of this study highlight the necessity of national FP programs and interventions that provide appropriate and accurate information about the advantages of small families^41,42^.

Women who have unintended pregnancies have a lesser possibility of getting high CCI scores, according to this study. This finding is consistent with earlier research conducted in Bangladesh^14^ that demonstrated that unplanned births were linked to reduced utilization of specific MNCH services and completion of CoCs for maternal health. This association could be explained by the fact that women who have unwanted pregnancies are less emotionally and financially prepared than women who have planned pregnancies for the rigors of pregnancy and childbearing. Previous studies from Bangladesh^14^ and other low resource countries^43,44^ found that women who have unplanned pregnancies are more likely to neglect themselves and the growing fetus during pregnancy, as well as the newborn and childcare following the pregnancy. Our research contributes to the corpus of knowledge about the effects of unwanted pregnancy on MNCH care in developing countries, and it will have significant consequences for the completion of the CoC for MNCH care.

There are some positive aspects to the analysis. First, our study is unique in that it tracked the level of CoC from pregnancy to childhood, as well as during reproductive healthcare, allowing the results to be utilized to track the amount of CoC improvement over time. Second, in addition to estimating AOR, we provided RII and SII estimates to analyze socioeconomic inequalities in CoC for MNCH care, as they are more robust indices of socioeconomic inequality than AOR. Finally, given the large representative sample size and high response rate, selection bias is unlikely to have influenced the results. The study's measurement bias is anticipated to be minimized due to the BDHS's utilization of qualified employees and validated questionnaires^7^.

When explaining our findings, some constraints must be acknowledged. First, CoC results relied heavily on self-reported impressions and recall, which can be influenced by social desirability and subject to misclassification. We chose to study women who had a live delivery in the three years before to the survey to mitigate the effects of this constraint. Second, due to the cross-sectional nature of the data, causal inference cannot be emphasized. The SES of a child, for example, was measured after the birth of the child. Measuring SES before and after childbirth could help researchers better understand the link between SES and CoC for MNCH care. Nonetheless, research suggests that these methods are just as reliable as prospective longitudinal surveys.

Finally, because such data were not collected in the BDHS 2017–2018, our adjusted regression models did not account for all potential contributing variables or barriers to care, such as a woman's history of or current issues that could influence her care-seeking behavior, nor did they account for a quality approach to measuring the level of CoC. Completing four or more ANC visits, for example, did not guarantee that a woman received all of the necessary ANC services. More studies should be conducted in an effort to establish a CoC assessment that focuses on the dimension of care quality.

Despite these flaws, this study has produced significant information on CoC throughout a woman's reproductive life cycle and its relationship to SES, which may be utilized to develop programs and policies to improve CoC for MNCH care in Bangladesh.

## Conclusions

The completion rate of the complete CoC for MNCH was exceedingly low, indicating that Bangladeshi women, newborns, and children are not getting the most out of current health care. SES was found to be one of the key drivers of completion of CoC for MNCH services, with participants in the high SES group having higher CCI values than those in the low SES group. When other sociodemographic variables are considered, women with a secondary or higher secondary education, as well as women who were exposed to mass media at least once a week, women who lived in an urban setting, women who had an intended pregnancy, and women with one parity, have a significant relationship with the likelihood of having high CCI scores. As a result of the findings, other sociodemographic factors must be considered in addition to SES in order to increase the CoC completion rate from pregnancy to childhood. Future longitudinal research will be required to investigate the impact of possible processes mediating the association between household SES and CoC for MNCH services.

The current findings emphasize the relevance of initiatives, programs, and policies aimed at raising comprehensive CoC completion rates among women in Bangladesh. In the Bangladesh National Strategy for Maternal Health 2019–2030^45^, the government of Bangladesh has already provided a number of strategic directions, including prioritizing reducing existing inequalities in accessing and utilizing MNCH services, in light of the low coverage of MNCH utilization in the country. As a result, the SES disparities in comprehensive CoC utilization identified in this study will be critical in determining priorities, developing national action plans, and making policy recommendations in maternal and child health to help Bangladesh make progress in reducing maternal and neonatal mortality and morbidity.

Microcredit, economic livelihoods, conditional cash transfers (CCTs), and voucher programs are examples of economic interventions^46,47^ that have the potential to increase comprehensive CoC completion among women with low SES. A voucher program in Bangladesh^45^ has been shown to be beneficial in improving CoC uptake among the impoverished SES group. Voucher schemes have been found to be effective in increasing reproductive health care and pregnancy follow-up for women from poor families in Cambodia^48^, India^49^, and Uganda^50^.

Considering other significant sociodemographic variables such as women's education, the government of Bangladesh may invest more in women's education, raising women's social position and increasing comprehensive CoC uptake. An integrated healthcare system that incorporates collaboration and teamwork at all levels, from the home to the hospital, is another critical component that can enhance comprehensive CoC uptake in Bangladesh. This concept of a home-to-hospital CoC has been demonstrated to be beneficial in Bangladesh in providing appropriate health care to women from low socioeconomic backgrounds^45^. The government should also take the advantage of mass media to favorably affect Bangladeshi women's comprehensive MNCH utilization.

Furthermore, making further progress in reaching a greater completion rate of complete comprehensive CoC for Bangladeshi women, particularly among women with unwanted pregnancies, necessitates considering community behavioral change and addressing the current workforce shortfall.

## Data Availability

The datasets used and analyzed during the current study are available from the Measure DHs website: https://dhsprogram.com/data/available-datasets.cfm.
